# Does the Netherlands comply with national law and Article 2 of human rights concerning deceased minors?

**DOI:** 10.1371/journal.pone.0332741

**Published:** 2025-09-17

**Authors:** Sophie C. T. van Rhenen, Tess M. Wemeijer, Cédrique A. S. Gregoire, Wilma L. J. M. Duijst

**Affiliations:** 1 Faculty of Medicine, University of Groningen, Groningen, The Netherlands; 2 Public Health Forensic Department, GGD IJsselland, Zwolle, The Netherlands; 3 Department of Criminal Law and Criminology, Faculty of Law, Maastricht University, Maastricht, The Netherlands; 4 Department of Radiology and Nuclear Medicine, Maastricht University Medical Centre, Maastricht, The Netherlands; Florida International University, UNITED STATES OF AMERICA

## Abstract

**Background:**

In the Netherlands, reporting the death of a minor to a forensic physician has been legally required since 2010. Additionally, the Postmortem Evaluation of Sudden Death in Youth (PESUDY) procedure can be initiated for unexplained natural deaths.

**Objective:**

This study assesses the reporting rate of deceased minors to forensic physicians, evaluates PESUDY submission rates, and explores reasons for declining PESUDY, under the European Convention on Human Rights (ECHR) and the Convention on the Rights of the Child (CRC) obligations.

**Participants and setting:**

This observational, retrospective study examined data on deceased minors in the Netherlands in 2022 and 2023.

**Methods:**

Data were collected from forensic registration databases, the Central Bureau of Statistics (CBS) and the PESUDY scientific committee. PESUDY submission rates were analyzed, along with reasons for declining the procedure.

**Results:**

The reporting rate of deceased minors to forensic physicians represented 69% in 2022 and 68% in 2023. The reporting rates for perinatal deaths and deaths outside this period were 47% and 96% in 2022, and 57% and 83% in 2023, respectively. The submission rate of PESUDY-eligible cases was 66% in 2022 and 68% in 2023. The reasons for not pursuing PESUDY were parental refusal without explanation (nine cases), and religious reasons (three cases). In 40 cases, there were no reasons documented.

**Conclusions:**

The reporting of deceased minors is not adhered to, with stagnating reporting rates. Additionally, the need for parental consent for post-mortem investigations hinders thorough examination, potentially obscuring cases of child abuse. This brings about nonconformity with the ECHR and CRC on the right to life.

## Introduction

The tragic event of a child dying suddenly and unexpectedly, creates interests that need to be addressed. A proper investigation into the cause of death needs to be done in order to rule out external causes such as child abuse, mourn loss, and eventually enhance public health.

From the perspective of human rights and specifically children’s rights, questions arise about this system of death investigation, specifically for minors [1]. The European Convention on Human Rights (ECHR) applies without discrimination on the basis of age. The right to life, enshrined in Article 6 of the Convention of the Rights of the Child (CRC) and Article 2 of the ECHR, not only protects but also encourages the state’s positive actions to ensure that children’s lives are protected and the causes of their deaths are established [2–4]. The European Council of Legal Medicine (ECLM) describes the sudden and unexpected death of a child as one of the situations in which at least an autopsy should follow [5]. The CRC is built on protecting children as they mature into adulthood [6,7]. As a result, measurements should be taken against any form of child abuse by including procedures to investigate and prevent such abuse [8].

Children’s rights are inextricably linked to the position of parents and/or other caregivers. For instance, rights such as the right to privacy and freedom of thought, conscience and religion may constitute a conflicting interest in the procedural obligation to investigate the cause of death. ECHR case law shows that investigating the cause of death can prevail over the interests of relatives, underlining the importance of effective death investigation [9]. Equally, even though a person’s rights expire after death, the duty to treat the body with respect is still present [10]. Depending on the circumstances of the case, parents’ beliefs (e.g., religion) may be of lesser interest. A careful balancing of interests is crucial [11].

Dutch law investigates death as follows: A physician can declare a natural death after performing an external examination of the body. If the physician is not convinced of a natural death, he shall further consult a forensic physician. In the event of a natural death, the deceased’s family can consent to an autopsy. In the event of an unnatural death (e.g., murder, accidents or suicide), the public prosecutor shall be informed so as to initiate a criminal investigation [12]. A criminal investigation includes post-mortem radiology and a forensic autopsy ordered by the public prosecutor [13]. In principle, this procedure is similar for adults and children. Since 2010, however, there is a legal obligation to contact a forensic physician in all deaths of minors, natural and unnatural [14]. The percentage of deceased minors reported to the forensic physician increased from 51% to 70% between 2010 and 2017, thereby not adhering to the 100% that is required by law [15–17].

Additionally, the possibility of a Postmortem Evaluation of Sudden Death in Youth (PESUDY) has been legally regulated since 2010 [17,18]. In the event of an unexplained death of a minor, the PESUDY procedure can be initiated to determine the cause of death. Furthermore, the minor must have been discharged from the hospital after birth to be included in the PESUDY procedure. The PESUDY’s initial hybrid medical and judicial procedure objective was primarily to rule out child abuse [19]. The current procedure is the result of the multi-year impulses to improve the post-mortem examination of minors, and its objective has been adjusted over time. Since 2017, the procedure has been offered to the deceased minor’s custodial parents, and can only be initiated at their explicit request to support their grieving process [20]. In most cases, the legal guardians are the biological parents. However, if legal custody has been assigned to others, they are the ones entitled to make decisions. In situations where multiple individuals share legal custody, they must reach a joint decision. If one of the legal guardians refuses consent for the PESUDY procedure, the procedure will not be initiated, in accordance with the physician’s oath to do no harm: the PESUDY procedure is always supplementary and never medically necessary. If only one person holds legal custody, they are authorized to make the decision independently [13,14,21,22]. Their wishes regarding death investigation (whether toxicology, radiology and/or an autopsy) are decisive and apply to any post-mortem examination. A procedure originally intended to detect abuses in the most vulnerable group in society, may now encounter worrying obstacles. Until now, no research has ever been conducted to determine whether the PESUDY procedure is offered in eligible cases, and the rate of parental decline [4].

We conducted this study to assess the reporting rate of deceased minors to forensic physicians after 2017, to examine the submission rate of eligible deceased minors for the PESUDY procedure, and to identify the reasons for declining the PESUDY procedure. Additionally, the study evaluates how these findings align with the international obligations outlined in the ECHR and the CRC regarding adequate and effective post-mortem examinations of children.

## Methods

### Study design

We conducted an observational, retrospective study by collecting and analyzing data on the deaths of minors in the Netherlands from January 1, 2022 to December 31, 2023.

### Ethics statement

This research has been assessed by the Faculty Niet-WMO Verplicht Research Ethics Committee of the Maastricht University, and is approved in writing under approval number: FHML-REC/2023/137.

### Data collection

All cases of deceased minors registered by the forensic physician in 2022 and 2023 in the Netherlands were requested from the Municipal Health Services (GGD) and the Forensic Physicians Rotterdam Rijnmond (FARR). All GGDs and FARR granted permission for the data exports from their registration systems called Formatus and MicroHIS, respectively. Moreover, national coverage of all registered deceased minors by forensic physicians was thereby achieved (S1 and S2 Appendices). Since the data were anonymized, no consent was required from the participants themselves and/or their guardians for accessing and utilizing the data. The data were accessed between March 18, 2024, and July 24, 2024. The authors did not have access to information that could identify individual participants during or after data collection.

Furthermore, the number of deceased minors in 2022 and 2023 in the Netherlands was requested and received from the Central Bureau of Statistics (CBS). Data about the perinatal mortality in 2022 and 2023 were available directly from the publicly available CBS ‘Statline’ database. 

The study population included all individuals under the age of 18 years old who died in the Netherlands in 2022 and 2023. Moreover, this study excluded neonates born at sooner than 24 weeks of gestation who died within 24 hours [23]. Perinatal mortality was defined as follows: All stillbirths at 24 weeks of gestation or later, and all live births that resulted in death within seven days postpartum.

Finally, the total number of PESUDY submissions in 2022 and 2023 was obtained from the national PESUDY scientific committee. To determine the PESUDY procedure submission rate, only the deaths that met the PESUDY criteria were included from the forensic registration databases: the forensic physician determined the cause of death as unexplained but natural, and the deceased minor was born at term and had been discharged from the hospital since birth.

### Data analysis

#### Primary outcome measures.

- The reporting rate of deceased minors to forensic physicians in the Netherlands in 2022 and 2023.
- The rate of eligible cases submitted for the PESUDY procedure in the Netherlands in 2022 and 2023.
- Reasons for declining the PESUDY procedure in eligible cases.

#### Secondary outcome measures.

- The reporting rate of perinatal deaths to forensic physicians in the Netherlands in 2022 and 2023.
- The reporting rate of deceased minors, excluding perinatal deaths, to forensic physicians in the Netherlands in 2022 and 2023.

## Results

### Reporting rate deceased minors

**Fig 1:**
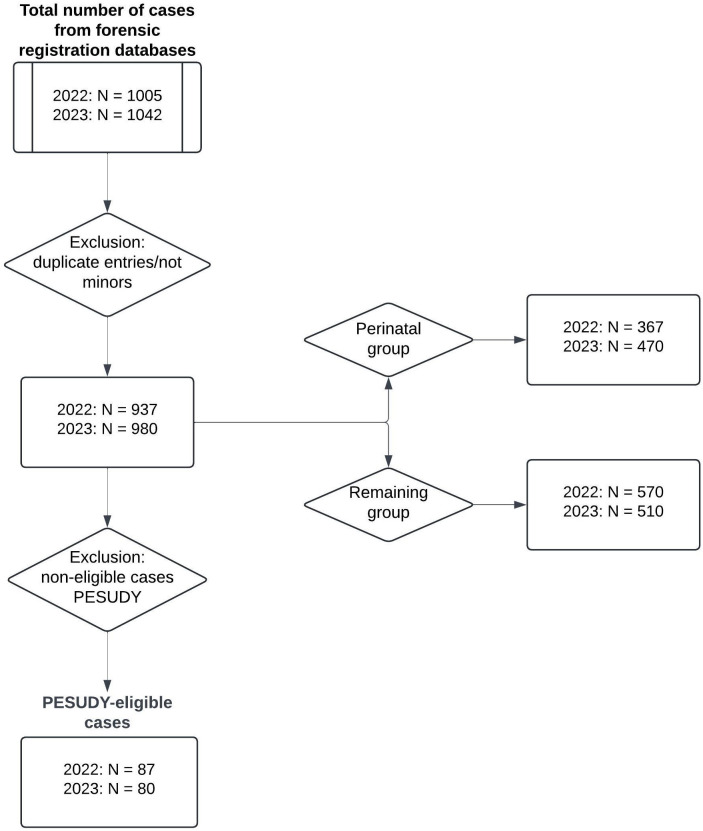
Case selection for determining the number of deceased minors registered by the forensic physician and PESUDY-eligible cases.

The data exports from the forensic registration databases contained 1005 cases from 2022 and 1042 cases from 2023. These include 63 cases from 2022 and 62 cases from 2023 deemed unsuitable to include in the results because of duplicate entries or definitional failure [23]. These cases were removed from the database, leaving 937 cases from 2022 and 980 cases from 2023 for analysis. In 2022, 367 of the 937 cases registered by the forensic physician fell under the definition of perinatal mortality. After excluding perinatal mortality from the forensic physician’s records, 570 cases remained. In 2023, 470 of the 980 cases fell under the definition of perinatal mortality, leaving 510 cases that fell outside the perinatal period (Fig 1).

In 2022, the CBS reported a total of 1365 deaths among minors, with 770 classified as perinatal mortality. Consequently, the remaining 595 deaths occurred beyond the perinatal period. In 2023, the CBS reported 1436 deaths among minors, with 821 classified as perinatal mortality. The remaining 615 deaths occurred beyond this period.

The total number of deceased minors per year in the forensic registration databases was compared with the data provided by CBS to identify any deaths not reported to the forensic physician. A reporting rate was calculated for each category by using the CBS data as the baseline (100%). The number of deaths in the forensic registration databases was then expressed as a percentage of this total. This analysis revealed a national reporting rate of 69% for all deceased minors in 2022 and 68% in 2023. Additionally, a reporting rate of 47% was observed for perinatal mortality and 96% for deaths among minors outside the perinatal period in 2022. For 2023, a reporting rate of 57% was found for perinatal mortality and 83% for deaths among minors outside this period (Table 1).

**Table 1:** Number of deceased minors reported to the forensic physician compared to CBS and the calculated reporting rate.

	Forensic physician	CBS	Reporting rate (%)
**2022, total**	937	1365	69
**2022, perinatal**	367	770	47
**2022, + 8d to 18yrs**	570	595	96
**2023, total**	980	1436	68
**2023, perinatal**	470	821	57
**2023, + 8d to 18yrs**	510	615	83

### PESUDY submission rate

The PESUDY-eligible cases were identified manually from the forensic registration databases. Cases lacking sufficient data to assess eligibility for the PESUDY procedure were excluded. This resulted in 87 eligible cases in 2022 and 80 in 2023. According to data from the PESUDY scientific committee, 57 cases in 2022 and 54 cases in 2023 were submitted for a PESUDY procedure.

The total number of eligible cases annually, as registered in the forensic registration databases, was compared with the number of PESUDY submissions reported by the PESUDY scientific committee. All eligible cases were considered the total (100%), and the number of submissions was expressed as a percentage of this total, representing the submission rate. This analysis revealed a national submission rate of 66% for 2022 and 68% for 2023 (Table 2).

**Table 2:** Number of PESUDY-eligible cases compared to the number of submitted cases and the calculated submission rate.

	Suitable cases	Submitted cases	Submission rate (%)
**2022**	87	57	66
**2023**	80	54	68

### Reasons for declining the PESUDY procedure

For all PESUDY-eligible cases, it was explored whether or not the case was submitted for the PESUDY procedure. When there was no record of submission for the PESUDY procedure, the reason for the decline was assessed. The reasons for declination were subsequently categorized, with a separate category for not mentioning the PESUDY procedure at all.

In 2022 and 2023, 167 PESUDY-eligible cases remained in the forensic registration databases. Of these, a total of 111 cases were submitted to the PESUDY procedure according to the data of the PESUDY Scientific Committee, leaving a gap of 56 suitable deceased minors where no PESUDY submission occurred. These 56 cases reflect the difference between the total number of eligible cases listed in the forensic databases and the number of actual submissions reported by the PESUDY Scientific Committee.

To investigate possible reasons for non-submission, only the forensic registration databases were used, as the PESUDY Scientific Committee does not register the cases in which the PESUDY procedure was declined; they only track the number of submissions. Based solely on the forensic registration databases, 52 cases were identified in which the PESUDY procedure was declined. For another 12 cases in the forensic registration databases, it remained unclear which had been submitted for the PESUDY procedure. These 12 ambiguous cases were excluded, as it could not be determined in which instances submission was declined and in which it was not.

In all suitable cases, comments on the PESUDY procedure and reasons for rejection were considered. In the majority of cases (40), it was unclear why the PESUDY procedure was not pursued. These cases were estimated as eligible for the PESUDY procedure, but the procedure was not mentioned anywhere in the case files. In nine cases, it was noted that the parents refused the PESUDY procedure without specifying the reasons. In the remaining three cases, religion was cited as the reason, with one case also mentioning the desire to keep the child at home as a second reason (Table 3).

**Table 3:** Reasons for opting out of the PESUDY procedure: frequency of occurrence.

Reasons	Number of cases in 2022, 2023
Not registered	40
Refusal by parents, no further explanation	9
Religion	2
Religion and wanting to keep the child at home	1

## Discussion

In the Netherlands, the legal obligation to report deceased minors to the forensic physician is not being sufficiently adhered to. Since 2016, the reporting rate has stagnated at around 70%. The reporting rate is lowest in the perinatal mortality group at 47% in 2022. In addition to cases that are legally required to be reported, there are also cases submitted to the forensic physician failing to meet the reporting criteria. Over a two-year period, we excluded 125 cases from the forensic registration databases. These cases highlight a lack of awareness, particularly among gynecologists, but also among pediatricians and general practitioners, regarding the legal definition of a minor. Furthermore, in many cases, the PESUDY procedure is not carried out even when the case is eligible. The reasons for refraining from this procedure are rarely documented. As a result, it is impossible to determine why the PESUDY procedure was declined. One possibility is that the legal guardians refused the procedure; another is that the option of undergoing the PESUDY procedure was never even discussed with them; an enormous missed opportunity. If the guardians did indeed refuse, it would be valuable to understand the underlying reasons for their decision. Therefore, it is advisable to improve documentation practices regarding whether or not a case is registered for the PESUDY procedure.

This study has a number of limitations. The national reporting rate may be underestimated, as forensic physicians may not have registered all reported cases. On the other hand, the reporting rate could be overestimated due to possible duplicate entries resulting from multiple registrations and postmortem activities in the system, combined with anonymized data. Furthermore, the CBS only includes data from deceased Dutch residents, while forensic physicians also record non-residents. Only children stillborn after 24 weeks of gestation or children beneath this threshold, but who have lived for 24 hours or more, need to be reported. In some cases, it was unclear whether neonates were born before or after 24 weeks of gestation and whether they lived longer than 24 hours. Every reported case was included. This may have led to a slight overestimation.

In addition, since the reporting rate of deceased minors is less than 100%, the forensic registration databases do not contain all cases of deceased minors. As a result, only a portion of these deaths has been assessed for eligibility for the PESUDY procedure. Therefore, the submission rate for the PESUDY procedure calculated in this study may be an overestimation. It is likely that few eligible cases are among the missing registrations, as a case is only eligible if it involves an unexplained, natural death. Most attending physicians are more likely to contact a forensic physician in cases of unexplained deaths than when the cause of death is known.

It is worthwhile to provide a more detailed explanation of the figures underlying the PESUDY non-submissions. The total of 56 non-submission cases results from subtracting the number of submissions, as recorded by the PESUDY Scientific Committee, from the total number of PESUDY-eligible cases identified in the forensic registration databases.

However, in exploring the reasons for non-participation, only the forensic registration databases were consulted, as the PESUDY Scientific Committee does not record non-submissions. This resulted in 52 cases in which no submission for the PESUDY procedure had occurred. In addition, 12 cases were identified in the forensic registration databases for which it remained unclear whether a submission had taken place. It is plausible that four of these cases were not submitted, which would account for the total of 56 non-submission cases. The remaining eight cases would, in that scenario, have been submitted.

The weakness of the PESUDY procedure is the fact that for performing the procedure permission of both the parents is needed. The effect of asking for the parents’ permission is that in about one third of a sudden unexpected death of a child no investigation is done. In recent years a few Dutch cases of sudden unexpected death, in which child abuse was the cause of death, came to the attention of the authorities by mere incidence [1]. To meet the requirements of the ECHR and the CRC, a post-mortem procedure should be carried out in all cases of sudden unexplained death of a child. It would be helpful when toxicological and radiological investigations were obligatory in every case of sudden unexplained death of a child. And a forensic autopsy should be carried out when this investigation led to clues about child abuse or a non-natural death. When no clues are found, the parents of the child should have the right to ask for an autopsy. By doing so, the rights of the child and the rights of the parents are respected.

## Conclusions

The Dutch system for post-mortem investigation in minors contains two major flaws. First, the duty to report every death of a minor to a forensic physician is not met in full and a controlling mechanism is lacking. This study shows that 14 years after installing this legal obligation, the reporting number is still as high (or low) as in 2017. Without a report to the forensic physician, no decision can be made about the need for a further post-mortem investigation. Second, the obligation to ask parents’ consent for a further post-mortem investigation (meaning anything more than external post-mortem investigation) is lacking. When no permission is given, post-mortem investigation is obstructed.

These two weaknesses of the Dutch system create a chance of missing death by child abuse. English researchers found that in the UK in about 5% of the post-mortem investigation of sudden death a violent death was found [24]. There is no reason to assume that the Netherlands numbers will differ. The Dutch system of post-mortem investigation creates a dark number about child abuse which should not exist.

Therefore we can only conclude that the Dutch system does not fully comply with ECHR and CRC standards regarding the right to life, which is the most fundamental right of every human being, regardless of how small that human may be or how imperceptible his voice is.

## Supporting information

S1 File Data exports from GGD. The inclusion criteria.
(PDF)

S2 File Data export from FARR. The inclusion criteria.
(PDF)

S1 Table Data export from CBS. The raw, unfiltered data.
(XLSX)

S2 Table Data export from GGD and FARR. The raw, unfiltered data.
(XLSX)

S3 Table Data export from PESUDY Scientific Committee. The raw, unfiltered data.
(DOCX)

## References

[1] Gregoire C, Duijst-Heesters W. De Nederlandse naleving van internationale verplichtingen inzake Nader Onderzoek naar de Doodsoorzaak van Kinderen. Ned Tijdschr Voor Mensenrechten. 2024;49(2). doi: 10.54195/ntm.19052

[2] Factsheet “Protection of Minors.” European Court of Human Rights. 2024.

[3] Nencheva *et al*. v Bulgaria. European Court of Human Rights. Sect. 4, 48609/06. 2023.

[4] Putte E, Rudolph M. Evaluatierapport NODOK-procedure ten behoeve van het Ministerie van Volksgezondheid, Welzijn en Sport. UMCU, Ministerie van Volksgezondheid Welzijn en Sport; 2018. pp. 17. Available from: https://www.tweedekamer.nl/downloads/document?id=6cb53a58-b4f9-4f57-a014-b1e4207d70bb&title=Evaluatierapport%20NODOK-procedure%20ten%20behoeve%20van%20het%20Ministerie%20van%20Volksgezondheid%2C%20Welzijn%20en%20Sport.pdf

[5] Recommendation No. R (99) 3, paragraph 2(b). Council of Europe; 1999 Feb p. paragraph 2(b).

[6] Civil Code (Netherlands), Art. 1:233. 2015.

[7] Convention on the Rights of the Child Art. 1. United Nations Nov 20, 1989.

[8] Convention on the Rights of the Child Art. 19. United Nations Nov 20, 1989.

[9] Solska and Rybicka v Poland. European Court of Human Rights. Sect. 1, paragraph 123, 30491/17 and 31083/17 Sep 20, 2018.

[10] Duijst-Heesters W, Naujocks T. Over lijken: de dood en daarna, vanuit juridisch-medisch perspectief. 1st ed. Maklu; 2015.

[11] Polat v Austria. European Court of Human Rights. Sect. 4, 12886/16 Jul 20, 2021.

[12] Duijst-Heesters W, Soerdjabalie V, Woudenberg C. De lijkschouw en sectie beschouwd. Nederlands Forensisch Instituut; 2016.

[13] Burial and Cremation Act (Netherlands), Art. 72 and 73. 2015.

[14] Burial and Cremation Act (Netherlands), Art. 10A. 2006. 2005.

[15] Dorn T, Slev V, Ceelen M, Edelenbos E, Soerdjbalie-Maikoe V, Stomp J, et al. Evaluatie NODO-meldplicht 2010 tot medio 2013: Overleden minderjarigen gemeld door behandelend artsen aan de gemeentelijk lijkschouwer. GGD Amsterdam; 2014.

[16] Dorn T, Slev V, Ceelen M, Edelenbos E, Reijnders U. Dood minderjarige steeds vaker gemeld. Ned Tijdschr Geneeskd. 2019. 31682092

[17] Handelingsprotocol “Nader Onderzoek naar de Doodsoorzaak van Kinderen” (NODOK). Nederlandse Vereniging voor Kindergeneeskunde (NVK), Ministerie van Volksgezondheid, Welzijn en Sport (VWS); 2016.

[18] Pries AM, Ruskamp JM, Edelenbos E, Fuijkschot J, Semmekrot B, Verbruggen KT, et al. A Systematic Approach to Evaluate Sudden Unexplained Death in Children. J Pediatr. 2024;264:113780. doi: 10.1016/j.jpeds.2023.113780 37852434

[19] NODOK procedure. In: Forensisch Medisch Genootschap [Internet]. [cited 10 Sep 2024]. Available from: https://www.forgen.nl/thema/2/nodok-procedure

[20] Staatscourant. Ministerie van Volksgezondheid Welzijn en Sport; 2017.

[21] Civil Code (Netherlands), Art 1:245. 2002.

[22] Civil Code (Netherlands), Art 1:247. 2023.

[23] Burial and Cremation Act (Netherlands), Art. 2. 2015.

[24] Williams T, Sleap P, Pease A, Fleming P, Blair P, Stoianova S, et al. National childhood mortality database; sudden and unexpected deaths in infancy and childhood. Bristol Medical school; 2022.

