# Supplementary material for: Does the Netherlands comply with national law and Article 2 of human rights concerning deceased minors?
Source: PLoS One. 2025 Sep 17;20(9):e0332741. doi: 10.1371/journal.pone.0332741 (PMC12443281; doi:10.1371/journal.pone.0332741)
Supplement: S1 File — The inclusion criteria. (PDF) [file pone.0332741.s001.pdf]

Export 1: postmortem examinations

All postmortem examinations of deceased individuals up to and including 17 years of age, conducted by a GGD forensic physician in 2022 and 2023. The following variables were included in the file:

- Year of death (2022, 2023);
- Age at the time of death in years, months, or days;
- Province of death;
- Examinations conducted during the postmortem examination;
- Manner of death (natural, unnatural);
- Category of cause of death (natural death, suicide, abuse, accident, medical intervention, traffic accident);
- Conclusion;
- Recommendation.

Export 2: deceased minor notifications

All reports of deceased individuals up to and including 17 years of age, reported to a GGD forensic physician in 2022 and 2023. The following variables were included in the file:

- Year of death (2022, 2023);
- Age at the time of death in years, months, or days;
- Province of death;
- Less than 24 weeks of gestation (yes/no);

- Lived longer than 24 hours (yes/no);
- Stillborn (yes/no);
- Examination by forensic physician (yes/no);
- Conclusion (unknown; unnatural death; natural death, cause known; natural death, cause unknown, further investigation by hospital; natural death, cause unknown, no further investigation by hospital).

Export 3: late termination of pregnancy

All late terminations of pregnancy reported to a forensic physician from the GGD in 2022 and 2023. The following variables were included in the file:

- Year of termination (2022, 2023);
- Number of weeks of gestation;
- Province where the termination occurred;
- Compatible with life (yes/no).
