# Supplementary material for: Does the Netherlands comply with national law and Article 2 of human rights concerning deceased minors?
Source: PLoS One. 2025 Sep 17;20(9):e0332741. doi: 10.1371/journal.pone.0332741 (PMC12443281; doi:10.1371/journal.pone.0332741)
Supplement: S2 File — The inclusion criteria. (PDF) [file pone.0332741.s002.pdf]

Export 1: deceased minors registered by FARR

All reports and postmortem examinations of deceased individuals up to and including 17 years of age, reported to or conducted by a forensic physician from FARR. The file contains two tabs, one for 2022 and one for 2023. The following variables are included in the file:

- Date of death;
- Episode;
- Sex;
- Date of birth;
- Age on the date of death in years;
- Cause of death/conclusion;
- Province of death;
- Additional tests (urine, toxicology);
- Category (deceased minor notification, PESUDY, medical intervention, murder/manslaughter, suicide, natural cause of death, accident);
- Less than 24 weeks of gestation (yes/no/unknown);
- Lived longer than 24 hours (yes/no);
- Stillborn (yes/no);
- Postmortem examination by a forensic physician (yes/no);
- Number of weeks of gestation;
- Compatible with life;
- Remarks.
