# Supplementary material for: Does the Netherlands comply with national law and Article 2 of human rights concerning deceased minors?
Source: PLoS One. 2025 Sep 17;20(9):e0332741. doi: 10.1371/journal.pone.0332741 (PMC12443281; doi:10.1371/journal.pone.0332741)
Supplement: S3 Table — The raw, unfiltered data. (DOCX) [file pone.0332741.s005.docx]

| NODOK procedures per provincie van overlijden | | |
| --- | --- | --- |
| **Provincie** | **2022** | **2023** |
| **Groningen** | 5 | 2 |
| **Friesland** | 1 | 2 |
| **Drenthe** | 0 | 1 |
| **Overijssel** | 3 | 2 |
| **Flevoland** | 0 | 1 |
| **Gelderland** | 8 | 10 |
| **Utrecht** | 7 | 3 |
| **Noord-Holland** | 10 | 9 |
| **Zuid-Holland** | 8 | 14 |
| **Zeeland** | 3 | 0 |
| **Noord-Brabant** | 2 | 2 |
| **Limburg** | 6 | 6 |
| ***Onbekend*** | *4* | *2* |
